# The energy sensor AMPK regulates Hedgehog signaling in human cells through a unique Gli1 metabolic checkpoint

**DOI:** 10.18632/oncotarget.7070

**Published:** 2016-01-29

**Authors:** Laura Di Magno, Alessio Basile, Sonia Coni, Simona Manni, Giulia Sdruscia, Davide D'Amico, Laura Antonucci, Paola Infante, Enrico De Smaele, Danilo Cucchi, Elisabetta Ferretti, Lucia Di Marcotullio, Isabella Screpanti, Gianluca Canettieri

**Affiliations:** ^1^ Department of Molecular Medicine, Sapienza University of Rome, 00161 Rome, Italy; ^2^ Department of Experimental Medicine, Sapienza University of Rome, 00161 Rome, Italy; ^3^ Center for Life Nano Science@Sapienza, Istituto Italiano di Tecnologia, 00161 Roma, Italy; ^4^ Istituto Pasteur, Fondazione Cenci-Bolognetti, Sapienza University of Rome, 00161 Rome, Italy

**Keywords:** Hedgehog, AMPK, Gli1, cancer metabolism, phosphorylation

## Abstract

Hedgehog signaling controls proliferation of cerebellar granule cell precursors (GCPs) and its aberrant activation is a leading cause of Medulloblastoma, the most frequent pediatric brain tumor. We show here that the energy sensor AMPK inhibits Hh signaling by phosphorylating a single residue of human Gli1 that is not conserved in other species.

Studies with selective agonists and genetic deletion have revealed that AMPK activation inhibits canonical Hh signaling in human, but not in mouse cells. Indeed we show that AMPK phosphorylates Gli1 at the unique residue Ser408, which is conserved only in primates but not in other species. Once phosphorylated, Gli1 is targeted for proteasomal degradation. Notably, we show that selective AMPK activation inhibits Gli1-driven proliferation and that this effect is linked to Ser408 phosphorylation, which represents a key metabolic checkpoint for Hh signaling.

Collectively, this data unveil a novel mechanism of inhibition of Gli1 function, which is exclusive for human cells and may be exploited for the treatment of Medulloblastoma or other Gli1 driven tumors.

## INTRODUCTION

Sonic Hedgehog (Shh) pathway is a critical regulator of embryonic development, cell proliferation and stem cell fate. In the cerebellum, Shh signaling promotes postnatal proliferation of cerebellar granule cell progenitors (GCPs) [1], and its deregulation at this level causes Medulloblastoma (MB), the most frequent brain malignancy of the childhood [2, 3].

Shh ligand binds to the inhibitory receptor Patched (Ptch), thus relieving its inhibitory effect on the transmembrane transducer Smoothened (Smo). These events initiate a cascade of intracellular processes that lead to the activation of the Gli transcription factors (Gli1, Gli2 and Gli3). A key event for Gli activation is the dissociation from the cytoplasmic inhibitor Sufu [4]. Activation of Gli promotes the execution of transcriptional programs, regulating cell proliferation, survival, metabolism and migration. The aberrant activation of Shh pathway observed in MB and other tumors is due to genetic mutations of pathway components or to autocrine or paracrine ligand-dependent mechanisms of activation [5].

The understanding of the key role of Hedgehog (Hh) signaling in tumorigenesis has prompted investigation, aimed at identifying molecules targeting this pathway. Different drugs have been generated and the majority of them are Smo inhibitors. However, these molecules are inactive in case of mutations occurring downstream of Smo (i.e. Sufu or Gli mutations). Furthermore, clinical trials in patients and animals with tumors driven by mutations of Ptch or Smo have shown that, after a good initial response, tumor cells quickly acquire resistance to Smo antagonists. For these reasons it is now believed that alternative approaches, preferably targeting Gli, are required [6, 7].

A few Gli inhibitors, acting directly or indirectly, have been generated in recent years and some of them have shown a good specificity and efficacy in preclinical trials. However, further pharmacological validations and studies are still required before these compounds can enter clinical trials. An alternative approach could be the research of drugs of known clinical efficacy with the ability to target Gli or its downstream-regulated pathways.

Recent findings have shown that, during Hh-dependent proliferation, Gli transcription factors regulate transcriptional programs in normal and tumor cells, switching their metabolism toward aerobic glycolysis and lipogenesis and increasing the utilization of glucose and lipids [8–10]. This suggests that the availability of these nutrients may represent a key determinant to favor or prevent Hh-driven cell proliferation. A key sensor and regulator of nutrient availability is the AMP kinase (AMPK), which consists of three different subunits: alpha, beta and gamma [11]. Under low nutrient conditions, the intracellular AMP/ATP ratio raises and activates AMPK through the binding of AMP to the gamma subunit and release of the alpha, catalytic subunit [12]. This activates a coordinated compensatory response, aimed at restoring the intracellular ATP levels through increased mitochondrial activity and biogenesis, activation of autophagy and inhibition of energy demanding processes, such as cell proliferation [13]. In this view, energy-demanding developmental programs, associated to rapid tissue growth and nutrient consumption, such as the Hedgehog-regulated processes, could be ideal targets to be turned off under low nutrient conditions.

We report here the identification of a novel mechanism of inhibition of the Hh signaling at a downstream level, by the energy sensor AMPK. Given the availability of drugs activating this kinase, these findings may have relevant implications for the treatment of MB and other tumors associated to aberrant activation of the Hh signaling.

## RESULTS

### AMPK inhibits Hh signaling only in human cells

To determine whether the intracellular energy sensing machinery and the developmental Hh signaling are functionally connected, we tested the effect of different AMP Kinase (AMPK) agonists in Hh-stimulated cells. To this end, we used the two commercially available Hh-responsive cells: mouse fibroblasts NIH3T3 [14] and human medulloblastoma DAOY cells [15, 16]. Both cell lines were treated at early (6 hours) and late (24 hours) time points with the Smo agonist Sag, which activates Hh signaling [17], monitoring the mRNA levels of Gli1, a standard Hh target gene.

We first measured the effect of three commonly used AMPK activators: AICAR, which is converted in cells into ZMP that binds the alpha subunit of AMPK in place of AMP [18]; 2-deoxyglucose (2DG) and Metformin, which block glycolysis and mitochondrial function respectively, thereby leading to elevations of the AMP/ATP ratio [19].

**Figure 1:**
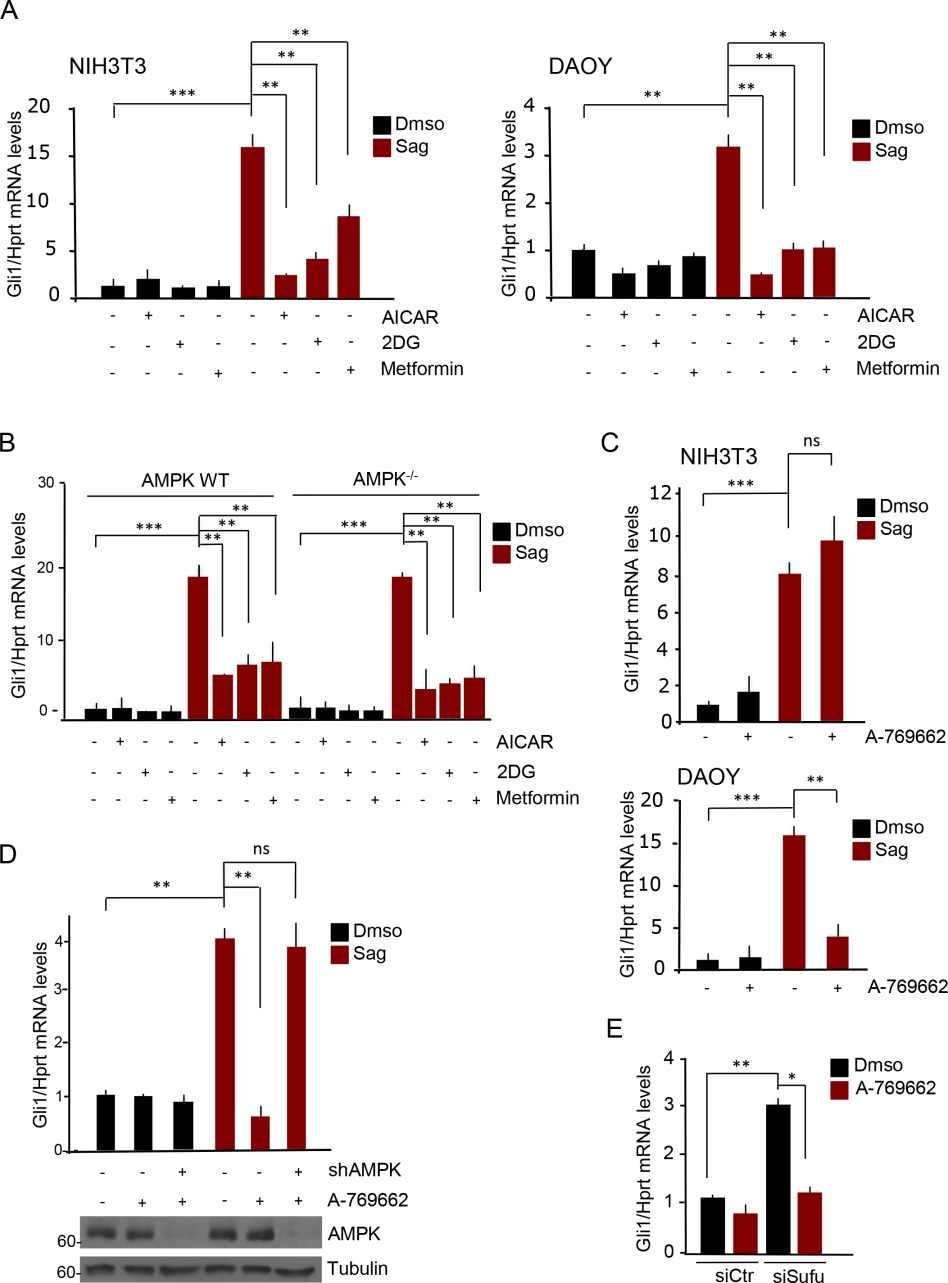
AMPK inhibits Hh signaling only in human cells (**A**) NIH3T3 (left) and human DAOY (right) cells were treated with Sag or Dmso, AICAR (2 mM), 2-deoxyglucose (2DG, 25 mM) or Metformin (5 mM) as indicated, for 6 hours. Levels of Gli1 mRNA were analyzed by quantitative PCR. (**B**) Wild type or AMPK^−/−^ MEF cells were treated with Sag or Dmso, AICAR (2 mM), 2-deoxyglucose (2DG, 25 mM) and Metformin (5 mM) as indicated for 6 hours. Gli1 mRNA levels were evaluated by quantitative PCR. The experiment was repeated three times. (**C**) NIH3T3 (top) and DAOY cells (bottom) were treated with Sag or Dmso, and with A-769662 (25 μM) for 24 hours. Gli1 mRNA levels were evaluated by quantitative PCR. (**D**) (Top) Quantitative real time PCR on AMPK-deficient DAOY cells. DAOY cells were infected with lentiviruses expressing shRNA targeting both α1 and α2 AMPK subunits (shAMPK) or with non-targeting shRNA. Cells were treated with Sag or Dmso and A-769662 (25 μM) for 24 hours as indicated. Gli1 mRNA levels were assessed. Knockdown efficiency was analyzed by immunoblotting with the indicated antibodies (bottom). (**E**) DAOY cells were transfected with siRNA against Sufu (siSufu), or with non-targeting siRNA (siCtr); cells were then treated with A-769662 (25 μM) or Dmso and Gli1 mRNA levels were measured by quantitative real time PCR. Results are expressed as mean and SD of three independent experiments, each performed in triplicate. *P < 0.05, **P < 0.01, ***P < 0.001 and ns (not significant) for the indicated comparisons.

Treatment with the three drugs robustly repressed Sag-induced Gli1 mRNA levels in both cell lines at both time points (Figure 1A, Supplementary Figure S1A).

To verify that this effect was mediated by AMPK we used the double AMPK alpha knockout MEF cells, lacking both alpha1 and alpha2 catalytic subunits of the kinase [20]. Interestingly, AICAR, 2DG and Metformin still inhibited the Hh-dependent transcriptional output, indicating that the observed inhibitory effect was independent of AMPK (Figure 1B). We then tested the effect of A-769662, a compound that was shown to bind the beta subunit and selectively activate AMPK, without off target effects [19, 21]. As shown in Figure 1C, incubation of Sag-treated cells with this drug for 24 hours did not have any significant effect in Sag-treated mouse fibroblasts, whereas it robustly inhibited the signaling in human DAOY cells. Knockdown of both alpha subunits of AMPK with shRNAs in DAOY cells prevented the A-769662 inhibition (Figure 1D), thus confirming that this effect was *bona fide* AMPK-dependent.

To study at what level of the Hh signaling AMPK exerts its inhibitory role, we tested the effect of A-769662 on Sufu-deficient DAOY cells. In the absence of Sufu, the transcriptional activity of the Gli transcription factors is upregulated with consequent increase of Gli-target gene expression, which is independent of upstream receptor activation [22].

Ablation of Sufu increased Gli1 mRNA levels and this effect was still inhibited by A-769662, indicating that AMPK exerts its inhibitory effect at downstream level (Figure 1E, Supplementary Figure S1B).

Thus, these data demonstrate that AMPK activation inhibits Hh signaling only in human cells, by targeting a downstream component of the pathway.

### AMPK phosphorylates Gli1 at Ser408

We tested the possibility that AMPK could directly phosphorylate human Gli1, Gli2 and Gli3 by performing an *in vitro* AMP kinase assay. We expressed human Flag-tagged Gli1-3 in HEK293T cells and performed Flag immunoaffinity purification, followed by the incubation of the eluted proteins with purified AMPK and ^32^P -labeled gamma ATP.

**Figure 2:**
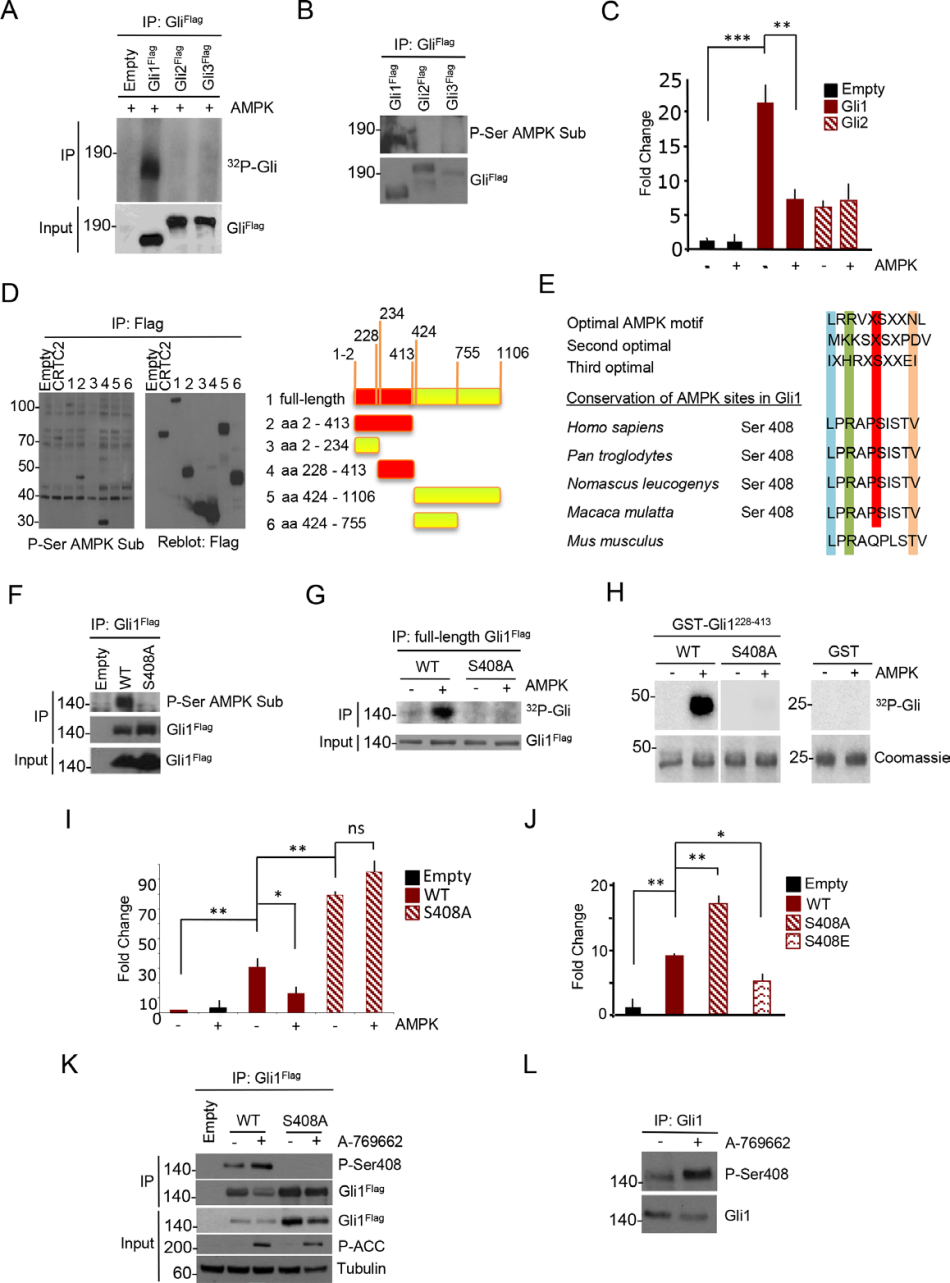
AMPK phosphorylates human Gli1 at Ser408 (**A**) Flag-Gli1, Flag-Gli2 and Flag-Gli3 proteins were expressed in HEK293T cells, and immunoprecipitated from whole cell lysates with Flag antibody. Eluted proteins were then incubated with catalytically active AMPK protein. ^32^P incorporation levels were assessed by autoradiography. Gli proteins expression was evaluated by western blot analysis with Flag antibody. (**B**) HEK293T cells were transfected with Flag-Gli1, Flag-Gli2 and Flag-Gli3 and overexpressed proteins were purified by immunoprecipitation. Phosphorylation was assessed by immunoblotting with anti-phospho serine AMPK substrate (P-Ser AMPK Sub) antibody. Filters were reprobed with Flag antibody to detect immunoprecipitated Gli protein levels. (**C**) Gli-Luc reporter assay showing the effect of AMPK overexpression on Flag-Gli1 and Flag-Gli2 transcriptional activity in DAOY cells. Results are expressed as Luciferase/Renilla fold change relative to control sample. (**D**) Left, western blot analysis of immunoprecipitates from HEK293T cells, transfected with plasmids encoding full-length Flag-tagged Gli1 or indicated fragments. Phosphorylation of the various Gli1 regions was assessed. Flag-CRTC2 was used as positive control. Right, schematic representation of Gli1 fragments. Red: phosphorylated fragments. (**E**) Protein sequence alignment of primates and murine Gli1, showing a conserved AMPK phosphorylation motif around Serine 408 (Ser408). Optimal AMPK motives are shown. (**F**) *In vivo* phosphorylation assay in HEK293T cells. Flag-tagged WT or S408A mutant Gli1 proteins were overexpressed and immunoprecipitated. Phosphorylation was assessed by immunoblot with anti-phospho serine AMPK substrate (P-Ser AMPK Sub) antibody. WT and mutant Gli1 protein levels in immunoprecipitated samples and cell lysates (Input) was carried out with Flag antibody. (**G**) Kinase assay on WT and S408A mutant Gli1 proteins, with or without active AMPK protein. Flag-Gli1 WT and S408A mutant were expressed in HEK293T cells and immunoprecipitated. ^32^P incorporation was revealed by autoradiography. Gli1 proteins expression was evaluated by western blot analysis. (**H**) *In vitro* AMPK-phosphorylation assay of GST alone, recombinant GST-Gli1 228–413 WT or S408A mutant. Incorporation of ^32^P was determined by autoradiography and the protein levels were detected by Coomassie blue staining. (**I**) Gli-Luc reporter assay showing the effect of AMPK on Gli1 WT and S408A mutant activity. Results are expressed as Luciferase/Renilla fold change relative to control sample. (**J**) Transcriptional activity of Gli1 WT versus non-phosphorylatable and phospho-mimetic mutants. Results are expressed as Luciferase/Renilla fold change relative to control sample. (**K**) HEK293T cells were transfected with Flag-tagged Gli1 WT and S408A mutant and treated with A-769662 (25 μM) for 1 hour. Cell extracts were immunoprecipitated with anti-Flag antibody and Serine 408 phosphorylation was revealed with a phospho-Serine 408 (P-Ser408) antibody. Immunoprecipitated Gli1 protein levels are shown. Western blotting of cell lysates (Input) was performed with Flag, phospho-ACC (P-ACC) and tubulin antibodies. (**L**) Serine 408 phosphorylation of endogenous Gli1 in DAOY cells. Cells were treated with A-769662 for 1 hour and Gli1 protein was immunoprecipitated from whole cell lysates. Immunoprecipitated Gli1 protein levels are shown. Results are expressed as mean and SD of three independent experiments, each performed in triplicate. *P < 0.05, **P < 0.01, ***P < 0.001 and ns (not significant) for the indicated comparisons.

As shown in Figure 2A, only Gli1 efficiently incorporated ^32^P in the presence of AMPK, whereas Gli2 and Gli3 did not. The same evidence was obtained *in vivo* in HEK293T cells, where the CAMKK2/AMPK axis is constitutively active and can be inhibited with the CAMKK2 inhibitor STO609 ([23] and Supplementary Figure S2A). After transfection of Flag-tagged Gli1-3 in these cells, IP and immunoblot with an antibody reacting against phosphorylated AMPK substrates, phosphorylation of Gli1, but not Gli2 or Gli3, was readily detected (Figure 2B, Supplementary Figure S2B).

Consistent with the observed inhibitory effect of AMPK agonists, AMPK overexpression inhibited Gli1, but not Gli2 transcriptional activity in luciferase assays (Figure 2C) in human DAOY cells. Also, expression of human Gli1 in mouse fibroblasts increased Gli-Luc reporter activity and this effect was significantly inhibited by A-769662, further demonstrating that AMPK specifically represses human Gli1 (Supplementary Figure S2C). Thus, AMPK phosphorylates and inhibits Gli1 function.

To map the AMPK phosphorylated residue/s, we tested the phosphorylation of several Gli1 fragments (Figure 2D) in HEK293T cells. Analysis of the phosphorylation status of the different mutants led to the identification of a 200 aa region, spanning from aa 228 to aa 413 that was efficiently phosphorylated (Figure 2D, Supplementary Figure S2D), whereas the other regions were not. Sequence analysis of the 228–413 region showed the presence of a consensus AMPK site surrounding the Serine 408 (Figure 2E). Interestingly, Ser408 residue is conserved only in primates, but not in mouse or other species, thus providing an explanation to the above-observed inhibitory effect of AMPK on Hh signaling in human, but not mouse cells.

We mutated Serine 408 to Alanine and observed that the phosphorylated Gli1 band completely disappeared in both *in vivo* and in *in vitro* kinase assays (Figure 2F–2H), indicating that Ser408 is the only AMPK phosphorylated residue of Gli1.

Notably, in Gli-Luc reporter assays the S408A mutant displayed higher transcriptional activity than WT Gli1 and was no longer inhibited by AMPK, supporting that this single residue is completely responsible of the effect of AMPK (Figure 2I). Further confirming this observation, a phosphomimetic S408E mutant showed significantly reduced transcriptional activity (Figure 2J).

To understand the physiological relevance of this modification, we generated and validated a rabbit antisera reacting against phosphorylated Gli1 Ser408 (Supplementary Figure S2E). Activation of AMPK with A-769662 increased ectopic and endogenous Ser408 phosphorylation (Figure 2K, 2L), but not of its mutant, and the AMPK inhibitor Compound C (CC) prevented this modification (Supplementary Figure S2F). Thus, AMPK phosphorylates Gli1 at Ser408, thereby inhibiting its activity.

### Ser408 phosphorylation promotes Gli1 degradation

We next sought to understand how Gli1 Ser408 phosphorylation exerts its inhibitory effect.

Previous studies on the mechanisms of AMPK-mediated inhibition have documented two recurrent mode of action upon phosphorylation of substrates: 1) binding to 14-3-3 proteins and cytoplasmic sequestration [24] and 2) targeting to proteasomal degradation and destabilization [25, 26].

**Figure 3:**
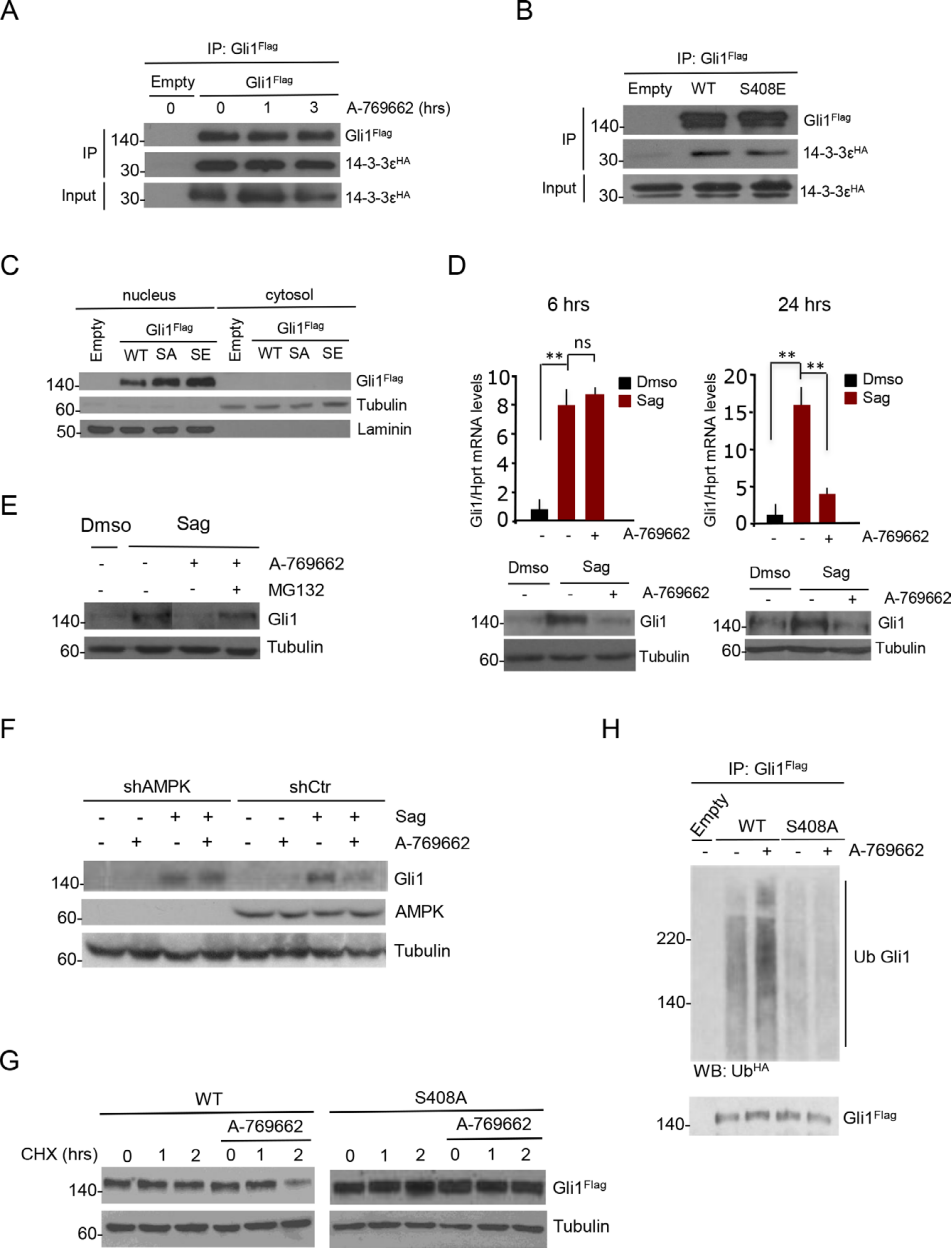
AMPK-mediated phosphorylation at Ser408 targets Gli1 to proteasomal degradation (**A**) Co-immunoprecipitation assay on DAOY cells transfected with plasmids expressing Flag-Gli1 and HA-14-3-3 epsilon. Cells were treated with or without A-769662 for the indicated times and Flag-Gli1 was immunoprecipitated. Binding was evaluated by western blotting of eluted proteins using Flag and HA antibodies. (**B**) As in A), DAOY cells were transfected with plasmids expressing Flag-Gli1 WT or S408A and S408E mutants, and HA-14-3-3 epsilon. After co-immunoprecipitation, immunocomplexes were detected with Flag and HA antibodies. (**C**) DAOY cells were transfected with Flag-WT and mutant Gli1 proteins. Nuclear and cytoplasmic fractions were analyzed by western blotting. Purity of nuclear and cytoplasmic fractions was evaluated by laminin and tubulin staining, respectively. (**D**) (Top) Quantitative real time PCR analysis of Gli1 mRNA levels on DAOY cells treated with or without Sag for 36 hours, and with or without A-769662 (25 μM) for 6 or 24 hours. (Bottom) Western blot analysis of cell lysates showing Gli1 and tubulin protein levels. (**E**) DAOY cells were treated with the proteasome inhibitor MG132 (50 μg/mL), and with the AMPK agonist A-769662 (25 μM), for 6 hours. The total cell lysates were analyzed by western blot using Gli1 antibody. Tubulin, loading control. (**F**) DAOY cells, infected with lentiviruses expressing shRNA targeting both α1 and α2 AMPK subunits (shAMPK) or with non-targeting shRNA (shCtr), were treated with Sag for 36 hours, and with A-769662 (25 μM) for 6 hours. Western blot analysis of cell extracts shows AMPK and tubulin levels. (**G**) DAOY cells were transfected 24 hours with Flag-Gli1 WT and S408A mutant and pre-treated with cycloheximide (CHX, 30 μM) for 20 minutes to inhibit protein synthesis. Cells were then incubated with or without A-769662 (12.5 μM) for the indicated time points. Western blots were performed on whole cell lysates using Flag antibody to detect Gli1 protein levels. Tubulin is shown as a loading control. (**H**) Flag-Gli1 WT and S408A mutant were co-transfected in DAOY cells with HA-Ub. Cells were pre-treated with MG132 (50 μg/mL) and incubated with A-769662 (25 μM) for 3 hours. Cellular lysates were immunoprecipitated with Flag antibody and ubiquitination revealed by HA-Ub western blotting. The blot was reprobed with Flag antibody to detect Gli1. Results are expressed as mean and SD of at least three independent experiments, each performed in triplicate. **P < 0.01 and ns (not significant) for the indicated comparisons.

Since an inhibitory binding between Gli1 and 14-3-3 epsilon proteins has been reported [27], we first tested the possibility that AMPK could increase this association by performing coimmunoprecipitation studies. We confirmed the association between Gli1 and 14-3-3, but did not observe any increased affinity following A-769662 treatment (Figure 3A). Consistently, neither the binding affinity for 14-3-3 (Figure 3B), nor the intracellular compartmentalization of S408E mutant differ from that of WT Gli1 (Figure 3C) thus ruling out this mechanism.

We next addressed the possibility that AMPK could regulate Gli1 turnover. DAOY cells were first treated with Sag to increase Gli1 protein levels and then incubated with A-769662 for 6 and 24 hours, monitoring Gli1 protein and mRNA levels. After 6 hours of incubation with A-769662, we observed that Gli1 protein levels were reduced, whereas the mRNA levels were not changed (Figure 3D, left). After 24 hours both mRNA and protein were decreased (Figure 3D, right), suggesting that, following AMPK phosphorylation, Gli1 is first destabilized and then the mRNA levels decrease as a consequence of its degradation. Both the proteasomal inhibitor MG132 (Figure 3E) and the lentiviral shRNA-mediated knockdown of AMPK (Figure 3F) prevented the A-769662 mediated downregulation of Gli1, indicating a proteasome-mediated, AMPK-dependent mechanism of degradation.

To confirm this observation, DAOY cells expressing WT or S408A mutant Gli1 were incubated for different time points with the protein synthesis inhibitor cycloheximide in the presence of A-769662 or vehicle. As shown in Figure 3G and Supplementary Figure S3A–S3B, the degradation rate of WT Gli1 was significantly enhanced by A-769662, while the AMPK-insensitive S408A mutant was more stable and not affected by the treatment. Consistently, A-769662 increased the ubiquitination of WT but not of Ser408A Gli1 mutant, whereas the AMPK inhibitor Compound C (CC) inhibited this modification (Figure 3H, Supplementary Figure S3C).

Collectively, these data demonstrate that AMPK phosphorylation at Ser408 promotes Gli1 turnover.

### Targeting Ser408 reduces proliferation of MB cells

Having found that AMPK inhibits Hh signaling via Gli1 phosphorylation, we next sought to understand if this mechanism could be exploited to limit Hh-dependent MB growth.

**Figure 4:**
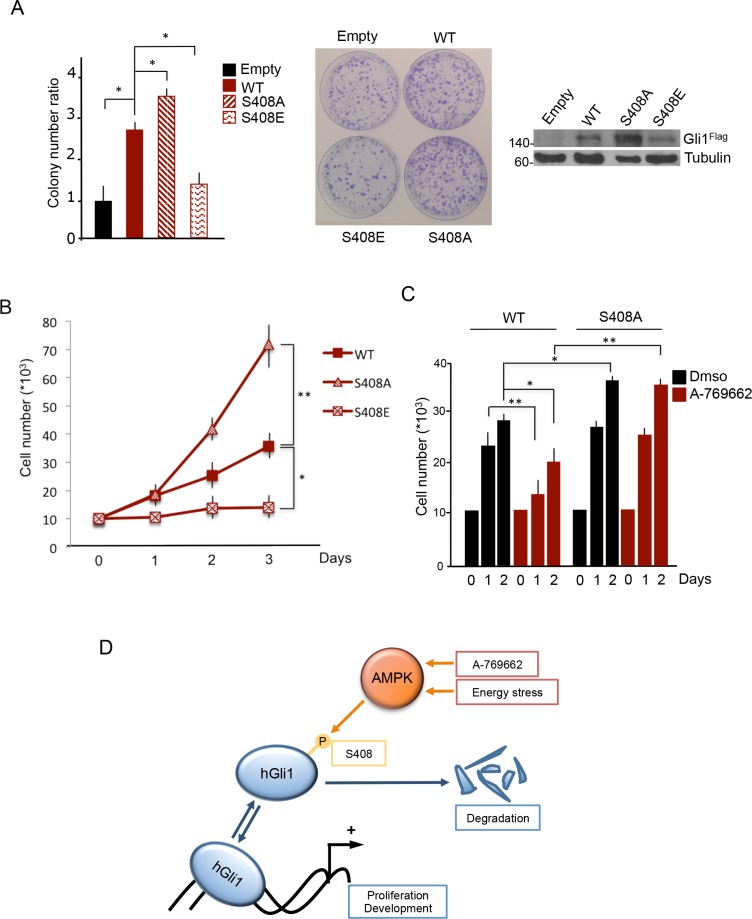
AMPK-mediated Ser408 phosphorylation inhibits Gli1 driven MB cell proliferation (**A**) (Left) DAOY cells stably expressing Flag-Gli1 WT, S408A and S408E mutants were seeded on 6-well plates and grown for 2 weeks. Colonies (larger than 1,5 mm) were counted. Middle, representative images of the colony assay. (Right) The amounts of the expressed Gli1 proteins were analyzed by immunoblotting with Flag antibody. Tubulin, loading control. (**B**) Proliferation rate of DAOY stable clones. Each clone was seeded in triplicate and counted at the indicated times. (**C**) As in B), cells were treated with A-769662 (25 μM) and counted at the indicated time points. (**D**) Model of the AMPK/Gli1 mediated mechanism of Hedgehog pathway regulation. Under normal conditions, Gli1 binds to Hedgehog target genes promoters and activates their expression to regulate proliferative and developmental processes. Activation of AMPK by drugs or energy stress phosphorylates Gli1, leading to its degradation. With this mechanism, AMPK inhibits Hedgehog pathway and Hedgehog-dependent events. Results are expressed as mean and SD of three independent experiments, each performed in triplicate. *P < 0.05 and **P < 0.01 for the indicated comparisons.

Since AMPK may inhibit cell proliferation with multiple mechanisms, recruiting different substrates [24], we addressed the specific involvement of Gli1 Ser408 phosphorylation by generating DAOY cell lines stably expressing Gli1 WT or the phosphorylation defective S408A or the phosphomimetic Ser408E mutants. As previously observed [28], stable expression of Gli1 caused a significant increase of colony formation and cell proliferation rate. Compared to cells expressing WT Gli1, the proliferation was significantly increased in cells expressing Ser408A mutant and decreased in those expressing Ser408E (Figure 4A, 4B), thus supporting the inhibitory role of Ser408 phosphorylation on Gli1-dependent proliferation. We next tested whether AMPK activation could counteract MB cell growth by specifically targeting Gli1 Ser408. Notably, as shown in Figure 4C, while treatment with A-769662 caused a significant inhibition of the proliferation rate in cells expressing WT Gli1, it failed to inhibit the proliferation driven by the S408A mutant. Therefore, this latter evidence demonstrates that the inhibitory effect of AMPK agonists on Gli1-driven proliferation requires the integrity of Ser408, which represents the key regulated checkpoint of this mechanism (Figure 4D). Similar data were obtained using the colon cancer cell line HT29 (Supplementary Figure S4), demonstrating that the Ser408-dependent proliferation effect is not cell-type specific, but rather Gli1-specific.

## DISCUSSION

Inhibition of Gli transcription factors is emerging as a potential valuable strategy for the treatment of tumors characterized by an “addiction” to Hh signaling. Indeed, the occurrence of resistance observed in patients treated with Smo antagonists has dampened the enthusiasm for this class of inhibitors and opened a new era of investigation, aimed at the identification of inhibitors acting at a downstream level.

In this report we demonstrate that AMPK is a potential weapon to achieve this goal, thanks to the ability of this kinase to directly phosphorylate and inhibit the downstream effector Gli1.

Interestingly, many drugs commonly used as AMPK agonists (e.g. AICAR, biguanides, 2DG) inhibit Hh signaling even in the absence of the kinase, as demonstrated by the persistence of inhibition in AMPK^−/−^ MEF cells. The off target effects of these compounds were already know and attributed to the regulation of other substrates, such as the mTORC regulator Rag GTPase or the mitochondrial respiratory-chain complex 1 [29]. While it is not surprising that the inhibition of protein synthesis or mitochondrial function may impact proliferation or survival in normal or tumor cells, the molecular basis of the AMPK-independent inhibition on Hh signaling exerted by these drugs is still an open issue.

The use of a specific AMPK agonist or knockdown approaches have allowed us to demonstrate that AMPK is a powerful inhibitor of Gli1 function, but only in human cells. This is due to the presence of a consensus AMPK site, Ser408, which is conserved only in primates, reasonably representing a mechanism acquired during evolution that enables the Hh pathway to directly respond to energy stress.

We demonstrate that activation of AMPK promotes Gli1 ubiquitination and proteasomal degradation. A similar mechanism of inhibition was described previously [26], where it was shown that AMPK phosphorylates the Glut1 inhibitor TXNIP, thereby targeting this protein for degradation with consequent increase of glucose influx. Interestingly, it was proposed that, upon phosphorylation, the ubiquitination/degradation of TXNIP is mediated by Itch, an E3 ubiquitin ligase. Since Itch has been shown to regulate Gli1 stability [30, 31] it is possible that this ligase is involved in the AMPK-dependent degradation of Gli1.

The ability of AMPK to regulate Hh signaling was previously observed in hepatocellular carcinoma cells [32], although the underlying mechanism was not understood.

A very recent report showed the ability of AMPK to phosphorylate and inhibit Gli1 [33]. However, the selectivity of the AMPK effect for only human Gli1 was not demonstrated, neither was reported the ability of the different drugs, such as AICAR and Metformin, to exert their inhibitory function even in the absence of AMPK.

Interestingly, in addition to Ser408 two other residues, not matching with any AMPK consensus sequence, were identified in that report: S102 and T1074. However, we could not detect in our assays any additional phosphorylated residue, beside Ser408. A possible explanation for this discrepancy could be that we did not overexpress the kinase together with Gli1, but used the constitutive activity of endogenous AMPK in HEK293T cells. Moreover, we could not detect any residual inhibitory activity of AMPK on Gli1 in the absence of S408, further supporting the functional importance of this site.

These aspects are relevant since the selectivity of AMPK for human Gli1 precludes further preclinical studies, evaluating the effect of selective AMPK agonists in mouse or other animal models of Hh dependent tumors. Studies with appropriate cellular models and clinical approaches are now required to evaluate the relevance of this mechanism for tumor treatment.

In conclusion, we have unmasked a functional connection between energy sensors and a tumor regulator, which pave the way to future studies aimed at validating the pathophysiological role of this mechanism in MB and other Hh-dependent tumors.

## MATERIALS AND METHODS

### Cell culture

NIH3T3 and HEK293T were cultured as previously described [34]. Human medulloblastoma DAOY cells were purchased from the American Type Culture Collection (ATCC, Manassas, VA, USA) and cultured in MEM with Earl's salts containing 10% FBS, 2% Penicillin-Streptomycin, 1% Glutamine, 1% Non Essential Aminoacids, 1% Sodium Pyruvate. AMPK double knockout (AMPK^−/−^) MEF cells were kindly provided by Benoit Viollet and cultured as described [20].

### Drugs and drug treatments

Sag was purchased from Adipogen Life Sciences (Liestal, Basel, Switzerland). For Hh pathway activation, DAOY cells were incubated overnight in serum-free medium, containing 1% BSA, and then exposed to Sag (200 nM) for the indicated time points.

AICAR was purchased from Cayman Chemicals, A-769662 from Tocris Bioscience, Metformin and 2-deoxyglucose from Sigma-Aldrich.

DAOY cells were pre-treated with MG132 (Calbiochem, Merk Group) to prevent protein degradation, and then incubated with A-769662. Cells were treated with cycloheximide (CHX, Sigma-Aldrich) and A-769662 as described in the text. The total cell lysates were analyzed by western blot using the indicated antibodies.

### Transfections, luciferase, binding assays, ubiquitination and kinase assays

Transfections and ubiquitination assays were performed as previously described [28, 35]. GST proteins were expressed in *E. coli* and purified with standard techniques. Luciferase assays were performed as described previously [28, 36].

For *in vivo* phosphorylation assay, HEK293T cells, transfected with full-length Flag Gli1 or Ser408A mutant, were lysed with the IP-Tris lysis buffer (50 mM TrisHcl pH 7.5, 1 mM EGTA, 1 mM EDTA, 0.5%Triton-X100, 10 mM sodium β-glycerophosphate, 1 mM Na_3_VO_4_, 50 mM NaF, 5 mM Na_4_P_2_O_7_, 0.1% (v/v) DTT, 0.15 M NaCl, 10% Glicerol, 0.1 mM PMSF), and immunoprecipitated with anti-Flag-Agarose (Sigma, A2220 IP 20 μl). After immunoblot, phosphorylation was detected with phospho-(Ser/Thr) AMPK Substrate antibody (#5759 Cell Signaling, 1:1000) or with the specific phospho-Serine 408 antibody (1:1000).

The *in vitro* ^32^P-labeled incorporation/radioactive phosphorylation assay was performed as previously described [37]. Recombinant Gli1 or full-length Flag Glis were incubated for 20 minutes at 30°C with 20 μl 1X Kinase Buffer (200 mM Tris pH 7.5, 50 mM β-glycerophosphate, 2 mM Na_4_P_2_O_7_) supplemented with 0.5 mM DTT, and 10 μl M-ATP buffer (300 μM ATP, 300 μM AMP, 66 mM MgCl_2_, 33 mM MnCl_2_), with or without 0.1U recombinant AMPK (Millipore, #14–840) and 10 μCi [γ-^32^P] ATP. The reaction was terminated by the addition of sample buffer (60 mM Tris, 2% SDS, 6% glycerol, 1% β-mercaptoethanol, and 0.002% bromophenol blue). Samples were separated by SDS-PAGE, and phosphorylation was detected by autoradiography.

### Quantitative PCR and RNA interference

Quantitative real time PCR was performed as previously described [28]. Briefly, total RNA was isolated with Trizol (Invitrogen) and reverse-transcribed with Superscript II reverse transcriptase and random hexamers (Invitrogen). A reaction mixture containing cDNA template, SensiFast Sybr Lo-Rox master mix (Bioline) and primer probes mixture was amplified using suggested Q-PCR thermal cycler parameters. Amplification primers are listed in Supplementary Table S1. Each amplification reaction was performed in triplicate and the average of the three threshold cycles was used to calculate the amount of transcript in the sample (using ViiA^™^ 7 software, Life Technologies, USA). Results were expressed as fold induction relative to control samples using the ΔΔCt method. Hprt was used as endogenous control.

siRNA transfections were performed by incubating DAOY cells with Hyperfect (Qiagen), according to the manufacturer's instructions. Briefly, siRNA were diluted in Optimem medium and supplied with the transfection reagent; the mixture containing the siRNA/Hyperfect complexes was then added to the cells, previously seeded in Optimem medium. After 6 hours, the transfection media was removed, and new fresh complete media was added. The day after transfection, a second transfection was performed and the cells were incubated for further 24 hours. Cells were then treated as indicated in the text. Human siSufu was purchased from Dharmacon (M-015382-00).

### Lentiviral-mediated shRNA knockdown

Subconfluent HEK293T cells were cotransfected by calcium phosphate precipitation with 20 μg of PLKO.1, 15 μg pCMV-R 8.74 and 10 μg pMD.G, to produce shAMPKα1 or shAMPKα2 or non-targeting lentiviruses. Supernatant was collected 24 and 48 hours later. Virus titer was determined using the HIV-1 p24 ELISA (NEK050B001KT, Perkin-Elmer), following manufacturer's instructions. pLKO.1 vectors used for lentiviral production were obtained by Sigma (MISSION shRNA SIGMA): Ampkα1 (TRC0000000861), Ampkα2 (TRC00000002171) and shc002 (control shRNA). To perform the lentiviral transduction, DAOY cells were seeded overnight in 12-well plates. The day after, 5 MOI of lentivirus were complexed with 5 mg/ml polybrene (Santa Cruz Biotechnology), added to the cells and left 24 hours before being removed and replaced with standard medium. Knockdown efficiency was monitored by AMPK western blotting.

### Cell proliferation and colony formation assays

To obtain DAOY stable lines, expressing wild type or mutant Gli1, cells were transfected with Lipofectamine and Plus reagent (Invitrogen), according to the manufacturer's recommendations.

After 48 hours from transfection, G418 (Cellgro) was added to the media, to a final concentration of 800 μg/ml. After 2–3 weeks of selection, colonies were picked and expanded. Throughout all the experiment, no changes in exogenous protein expression level, were detected.

Stable lines were then used to perform cell proliferation and colony formation assays [36]. For proliferation assays, 3 × 10^4^ cells were seeded in each 12 Multiwell plate well; cell proliferation was assayed every day through Trypan Blue cell count for three consecutive days. Each experimental point was performed in triplicate and each experiment was performed at least three times. To perform colony-formation assays, DAOY stable lines were detached, diluted to a concentration of 2 × 10^2^ for each 60 mm dish, and grown with G418 for 10–14 days to allow colony formation. Colonies were stained with Coomassie Brilliant Blue, and colony numbers and sizes were measured. Each experimental point represents a triplicate and the results shown are representative of at least three independent assays.

### Western blotting and immunoprecipitation assays

Lysates where separated by SDS–polyacrylamide gel electrophoresis, followed by subsequent western blot analysis. Gels were blotted on Protran Nitrocellulose Hybridization Transfer Membrane (PerkinElmer) and incubated with the indicated primary antibody, then with horseradish peroxidase-coupled secondary antibody. Detection of the horseradish peroxidase signal was performed using Western Lightning Plus ECL (PerkinElmer) according to the manufacturer's protocol.

Densitometry was performed on acquired images using ImageJ software (Rasband, W.S., ImageJ, U. S. National Institutes of Health, Bethesda, Maryland, USA, http://imagej.nih.gov/ij/, 1997–2015). Immunoprecipitation assays were performed as previously described [28].

### Cellular fractionation

Cellular fractionation was performed according to a previous report [38] with some modifications. Cellular pellets were resuspended in 100 μl buffer A (10 mM Hepes pH 7.4, 10 mM KCl, 10 mM NaCl, 0.1 mM EDTA, 0.1 mM EGTA, 1 mM DTT, 0.5 mM PMSF). After 15 minutes of incubation, 6 μl of NP40 (0.6%) were added and lysates were vortexed 10 sec and centrifuged at 11,000g for 30 sec. The supernatant containing the cytoplasmatic extract was centrifuged at 11,000g for 20 minutes, while nuclear pellets were washed in 500 μl of Buffer B (20 mM Hepes pH 7.4, 20% Glycerol, 100 mM KCl, 1 mM EDTA, 1 mM DTT, 0.5 mM PMSF, 10 mg/mL leupeptin), incubated for 15 minutes on ice and, after addition of 1% NP40, vortexed for 10 sec and centrifuged at 11,000 g for 30 sec. Nuclear pellets were resuspended in 50 μl of Buffer C (20 mM Hepes pH 7.4, 20% Glycerol, 400 mM NaCl, 1 mM EDTA, 1 mM EGTA, 1 mM DTT, 0.5 mM PMSF, 10 mg/mL leupeptin), incubated on ice for 20 minutes, vortexed, and centrifuged at 13,000 g for 10 minutes.

### Antibodies

The following antibodies were used: Gli1 (#2553S Cell Signaling, 1:1000), phospho-(Ser/Thr) AMPK Substrate (#5759 Cell Signaling, 1:1000), phospho-ACC (#3661S Cell Signaling, 1:2000), AMPK (07–181 Millipore, 1:1000), Sufu (#C8IH7 Cell Signaling, 1:1000), actin (sc-1616 SantaCruz 1:1000), tubulin (sc-8035 SantaCruz, 1:1000), laminin (sc-29012 SantaCruz, 1:1000), phospho-Serine 408 (Eurogenetec, 1:1000), HA-probe (F-7) HRP (sc-7392 SantaCruz, 1:1000), anti-Flag-M2 (F1804 Sigma, 1:1000), anti-Flag-M2 Peroxidase (A8592 Sigma, 1:5000).

### Plasmids and site-directed mutagenesis

12xGli-Luc, TK-Renilla, PGEX4T1-Gli1 228–413, full-length PCDNA3Flag-Gli1 and mutant plasmids, PCDNA3Flag-Gli2, PCDNA3Flag-Gli3, PCDNA3Flag-CRTC2, HA-Ub were previously described [28, 34, 36, 37]. HA-14-3-3 epsilon and Myc-AMPKα2 plasmids were purchased from Addgene.

Gli1 S408A and S408E mutants were obtained using QuickChange II XL Site-Directed Mutagenesis Kit (Stratagene/Agilent^®^). Primers sequences used for mutagenesis reaction are listed below:

Human Gli1 S408A Fw: 5′- CTGCCTCGGGC ACCAGCCATTTCTACAGTGGAG -3′;

Human Gli1 S408A Rw: 5′- CTCCACTGTAGAAAT GGCTGGTGCCCGAGGCAG -3′;

Human Gli1 S408E Fw: 5′- CTGCCTCGGGCACCA GAGATTTCTACAGTGGAG -3′;

Human Gli1 S408E Rw: 5′- CTCCACTGTAGA AATCTCTGGTGCCCGAGGCAG -3′.

### Statistical analysis

Statistical analysis was performed using StatView 4.1 software (Abacus Concepts, Berkeley, CA). Data were expressed as mean +/− SD, and statistical differences between the means were analyzed by the Mann-Whitney *U* test for non-parametric values. *P* < 0.05 was considered statistically significant.

## SUPPLEMENTARY MATERIALS FIGURES AND TABLE


