# Supplementary material for: The energy sensor AMPK regulates Hedgehog signaling in human cells through a unique Gli1 metabolic checkpoint
Source: Oncotarget. 2016 Jan 29;7(8):9538–49. doi: 10.18632/oncotarget.7070 (PMC4891058; doi:10.18632/oncotarget.7070)
Supplement: Supplementary file 1 [file oncotarget-07-9538-s001.pdf]

The energy sensor AMPK regulates hedgehog signaling in human cells through a unique Gli1 metabolic checkpoint

Supplementary Materials

MATERIALS AND METHODS

Cell culture and drug treatments

Human colon adenocarcinoma HT29 cells were obtained from Sigma-Aldrich and cultured in DMEM supplemented with 10% FBS, 2% Penicillin-Streptomycin, 1% Glutamine. Compound C (CC, Calbiochem, Merk Group) and STO-609 (Calbiochem, Merk Group) treatments were performed as described in the text.

Alkaline Phosphatase treatment of Gli1

Alkaline phosphatase was used to dephosphorylate Gli1. Flag-Gli1 was expressed in HEK293T cells, immunoprecipitated and incubated for 1 hour at 37°C with 10 U of Calf Intestinal alkaline Phosphatase (CIP, New England Biolabs), following manufacturer's instructions.

Kinase assay

Recombinant Gli1 228–413 was incubated for 20 minutes at 30°C with 20 µl Kinase Buffer (200 mM Tris pH 7.5, 50 mM β-glycerophosphate, 2 mM $Na_4P_2O_7$), supplemented with 0.5 mM DTT, and 10 µl M-ATP buffer containing 300 µM ATP, 66 mM $MgCl_2$, 33 mM $MnCl_2$, or 1X M-ATP buffer containing 300 µM ATP and 300 µM AMP, 66 mM $MgCl_2$, 33 mM $MnCl_2$ with or without 0.1 U recombinant AMPK and 10 µCi [Y-^{32}P] ATP. Gels were stained with Coomassie blue and ^{32}P incorporation was revealed by autoradiography.

Generation of pSer408 Gli1 antibody

The antibody against Gli1 Ser408 was generated by Eurogentec. KLH-coupled peptide H- GPLPRAP-S(*P03H2*)-ISTVE-NH2 was used to immunize rabbits. After four immunization boosts, sera were collected and antiserum analysis was performed by indirect ELISA.

Serum from the best responding rabbit was purified by double cross-affinity purification chromatography: the first with the modified, the second with the non modified peptide.

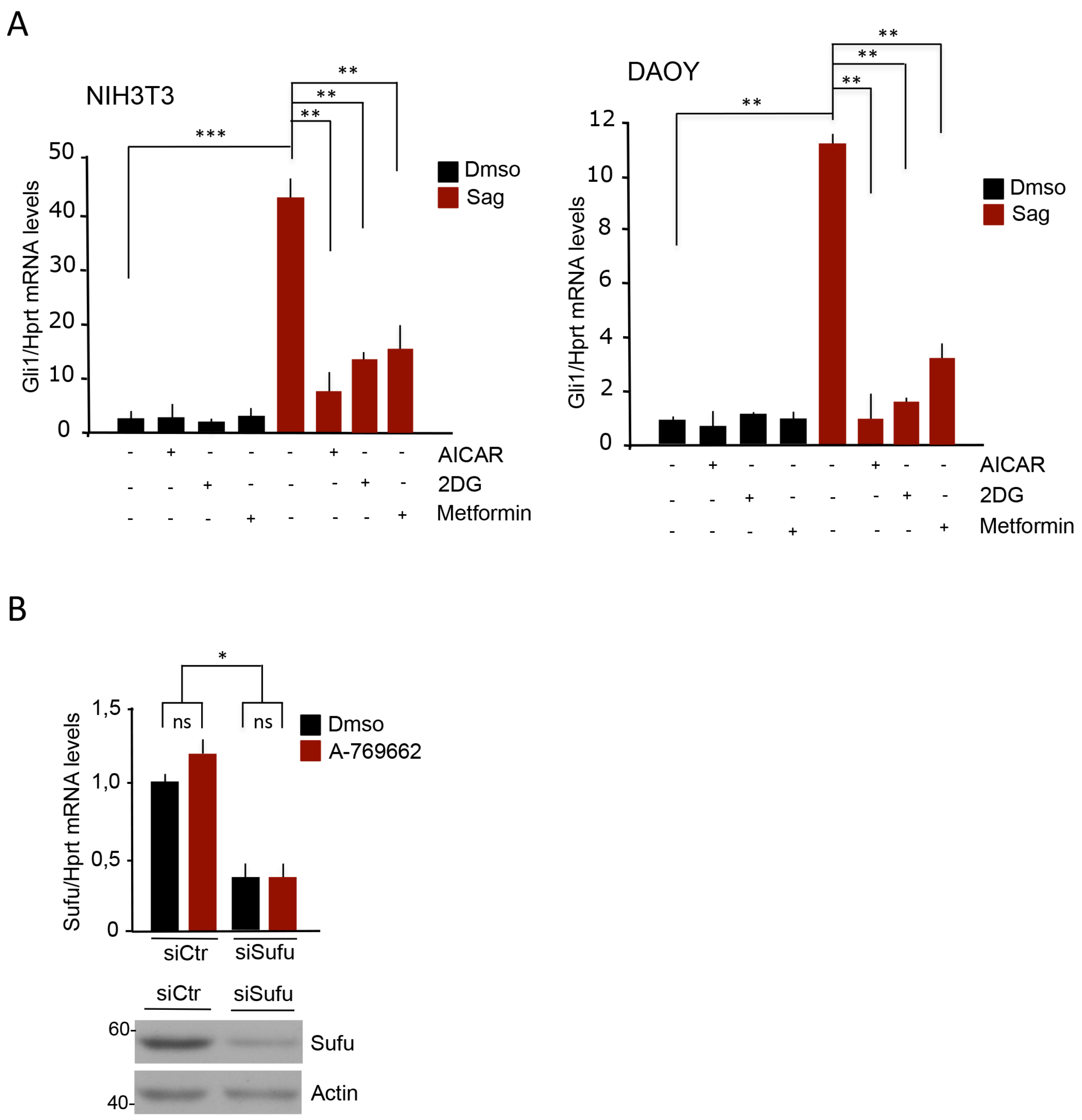

Supplementary Figure S1: (**A**) NIH3T3 (left) and human DAOY (right) cells were treated with Sag or Dmso, AICAR (2 mM), 2 deoxyglucose (2DG, 25 mM) or Metformin (5 mM) for 24 hours as indicated. Levels of Gli1 mRNA were analyzed by quantitative real time PCR and normalized by Hprt mRNA levels. (**B**) Sufu mRNA (top) and protein (bottom) levels normalized by Hprt and actin respectively in DAOY cells transfected with siRNAs targeting Sufu (siSufu) or control (siCtr). Results are expressed as mean and SD of three independent experiments, each performed in triplicate. $*P < 0.05$, $**P < 0.01$, $***P < 0.001$ and ns (not significant) for the indicated comparisons.

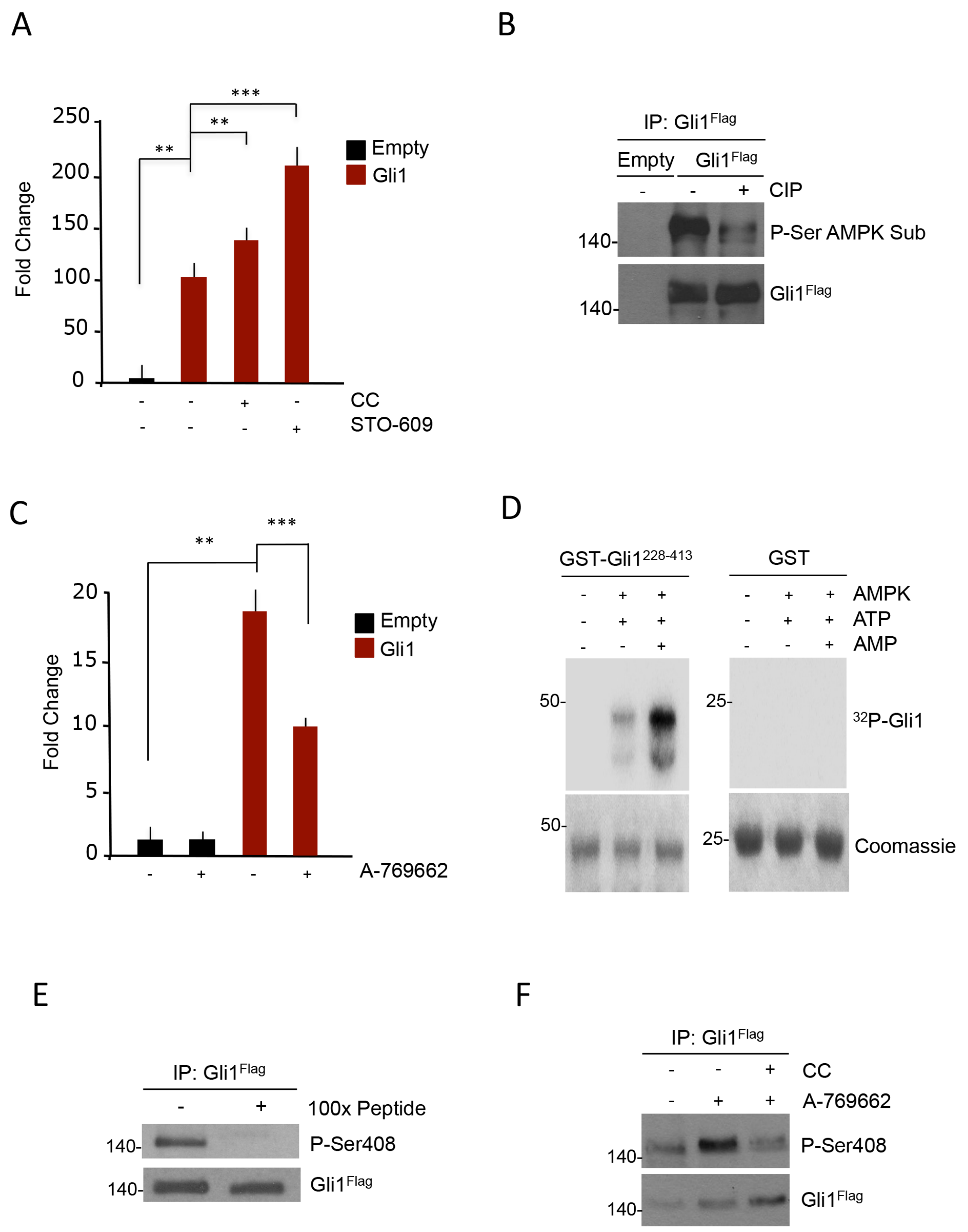

Supplementary Figure S2: (**A**) Effects of AMPK inhibition on Flag-Gli1 transcriptional activity in HEK293T cells, measured as Luciferase/Renilla ratio. Cells were treated with Compound C (CC, 20 μM) or ST0-609 (20 μM) for 4 hours. Results are expressed as fold change relative to the control sample. (**B**) Western blot analysis of immunoprecipitated Flag-tagged Gli1 in HEK293T cell extracts after dephosphorylation by calf-intestinal alkaline phosphatase (CIP, 10 U) for 1 hour. Gli1 phosphorylation was detected with anti-phospho serine AMPK substrate (P-Ser AMPK Sub) antibody. Filters were reprobed with Flag antibody to assess immunoprecipitated Gli1 protein levels. (**C**) Luciferase reporter assay in NIH3T3 cells transfected with human Gli1 plasmid, and treated with A-769662 (25 μM) for 8 hours. Results are expressed as fold change relative to control samples. (**D**) *In vitro* kinase assay using active AMPK and GST-Gli1 fragment (228–413) or GST alone. Reactions were performed in the presence of [y-^{32}P] ATP (10 μCi), or ATP and AMP (300 μM), to confirm the specificity of AMPK. Incorporation of ^{32}P was determined by autoradiography and the protein levels were detected by Coomassie blue staining. (**E**) The antibody specificity for phosphorylated Gli1 Ser408 was tested by immunoprecipitation and western blotting. Flag- Gli1 WT was transfected in HEK293T cells and immunoprecipitated. Filters were incubated with 5 mg/ml Gli1 phospho-Serine 408 (P-Ser408) antibody with or without 100 fold molar excess of immunogenic peptide. Immunoprecipitated Gli1 protein levels are shown. (**F**) Effects of the AMPK inhibitor Compound C (CC) on A-769662-induced Gli1 phosphorylation. HEK293T cells were transfected with plasmid encoding Flag-tagged Gli1 and pretreated with Compound C (CC, 20 μM) for 20 minutes before incubation with A- 769662 (25 μM) for 1 hour. Cell extracts were immunoprecipitated with Flag antibody and Gli1 phosphorylation was detected by western blot with phospho-Serine 408 (P-Ser408) antibody. Filters were reprobed with Flag antibody to detect immunoprecipitated protein levels. ***P* < 0.01, ****P* < 0.001 for the indicated comparisons.

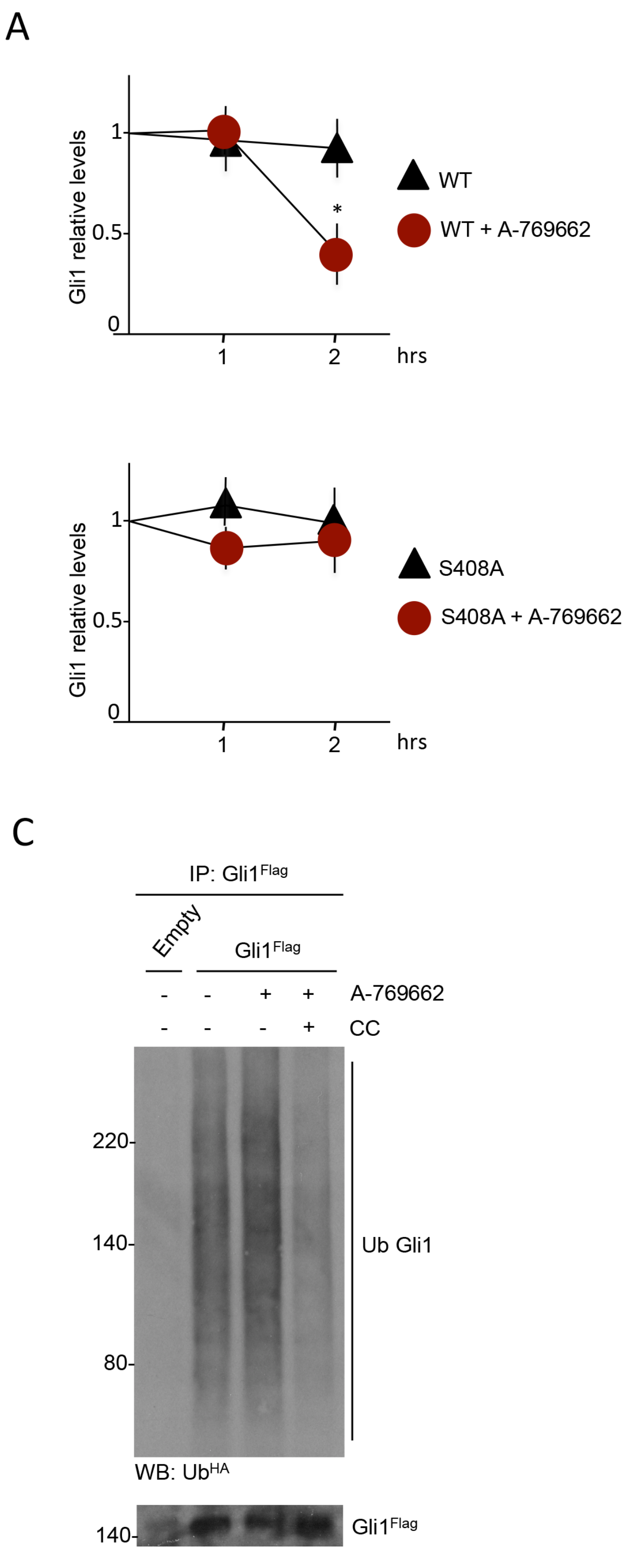

Supplementary Figure S3: (**A**) Densitometric analysis of samples from Figure 3G. WT and S408A protein levels are shown, after normalization with tubulin. (**B**) (Top) DAOY cells were transfected with Flag-Gli1 WT and S408A mutant and treated with cycloheximide (CHX). Lysates were harvested at the indicated time points and probed with Flag and tubulin antibodies. (Bottom) Densitometric analysis of WT and S408A protein levels after normalization with tubulin. Results represent the average +/− SD of three independent experiments, each performed in triplicate. (**C**) Ubiquitination assay in Flag-Gli1 and HA-Ub expressing DAOY cells. Cells were pretreated with Compound C (CC, 20 µM) for 20 minutes, and A-769662 (25 µM) was added to the culture medium for additional 3 hours. Lysates were immunoprecipitated with anti-Flag antibody and probed with anti-HA antibody. Gli1 levels in the immunoprecipitates are shown. Experiments were carried out in the presence of MG132 (50 µg/mL). $^*P < 0.05$ for the indicated comparisons.

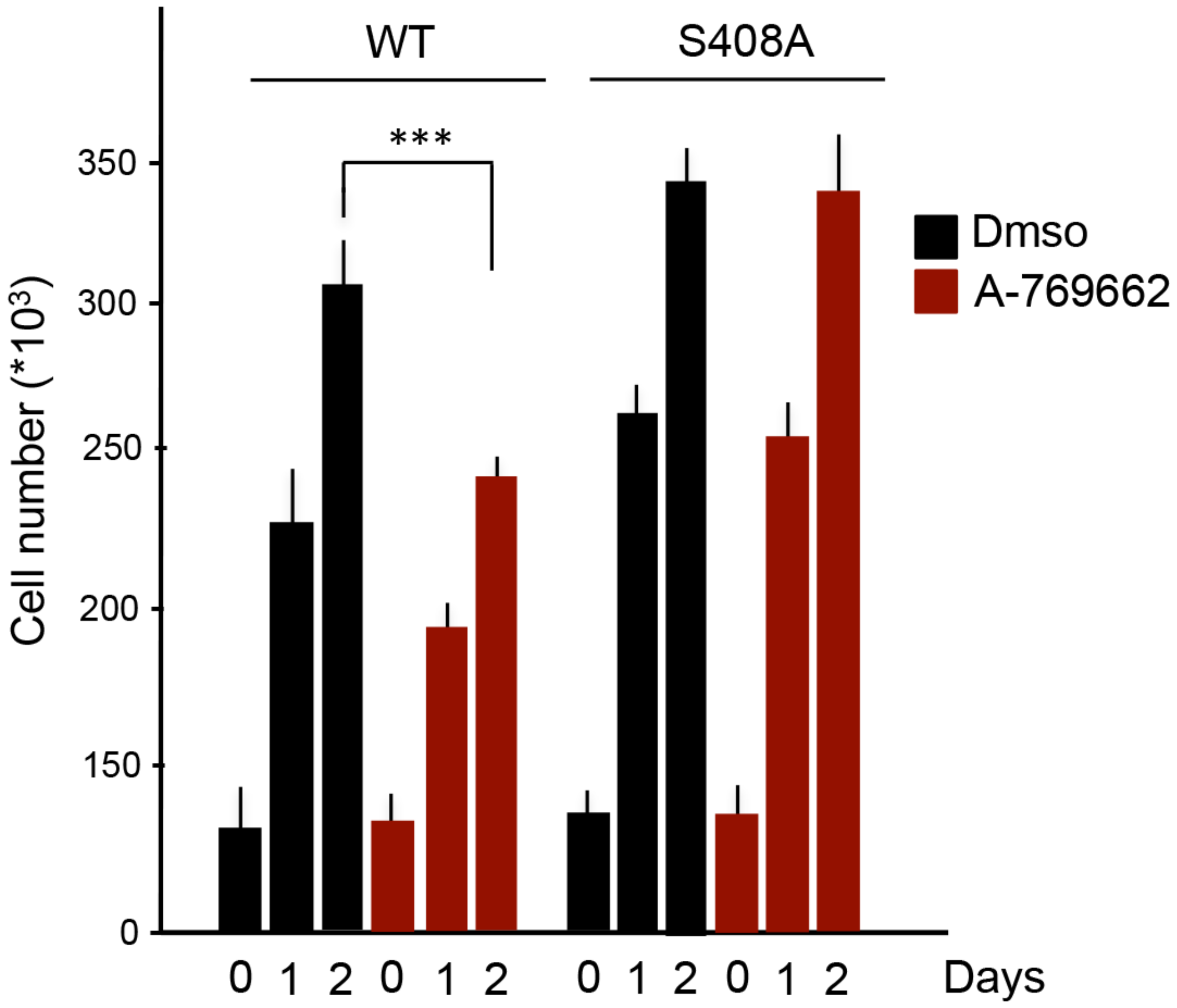

Supplementary Figure S4: Proliferation rate of HT29 cells stably expressing Gli1 WT and Gli1 S408A vectors. Clones were seeded in triplicate and treated with A-769662 (25 µM). Cells were counted at the indicated time points. ***$P < 0.001$ for the indicated comparisons.

Supplementary Table S1: Quantitative real time PCR oligonucleotide sequences

Sybr Green designed oligonucleotides

Human Gli1	Fw GGTGGTTCACATGCGCAG
	Rev GGTGCGTCTTCAGGTTTTCG
Human Hprt	Fw CGTCTT GCTCGAGAT GT GAT G
	Rev GCACACAGAGGGCTACAATGTG
Mouse Gli1	Fw AGACCAGCAGCTGCACTGAA
	Rev TGGCAGGTTGCACGTGGTC
Mouse Hprt	Fw GCTTCCTCCTCAGACCGCTT
	Rev GGTCAT AACCTGGTT CAT CATCG

Sybr Green oligonucleotide sequences for the indicated genes.
